# Metabolic pathway assembly using docking domains from type I *cis*-AT polyketide synthases

**DOI:** 10.1038/s41467-022-33272-2

**Published:** 2022-09-21

**Authors:** Xixi Sun, Yujie Yuan, Qitong Chen, Shiqi Nie, Jiaxuan Guo, Zutian Ou, Min Huang, Zixin Deng, Tiangang Liu, Tian Ma

**Affiliations:** 1 CAS Key Laboratory of Quantitative Engineering Biology, Shenzhen Institute of Synthetic Biology, Shenzhen Institute of Advanced Technology, Chinese Academy of Sciences, 518055 Shenzhen, China; 2 Key Laboratory of Combinatorial Biosynthesis and Drug Discovery, Ministry of Education and School of Pharmaceutical Sciences, Wuhan University, 430072 Wuhan, China; 3 State Key Laboratory of Microbial Metabolism, Joint International Research Laboratory of Metabolic & Developmental Sciences, and School of Life Sciences and Biotechnology, Shanghai Jiao Tong University, 200030 Shanghai, China; 4 Zhongnan Hospital of Wuhan University, School of Pharmaceutical Sciences, Wuhan University, 430071 Wuhan, China; 5 Hesheng Tech, Co., Ltd, 430073 Wuhan, China; 6 TaiKang Center for Life and Medical Sciences, Wuhan University, 430072 Wuhan, China

**Keywords:** Metabolic engineering, Biocatalysis, Applied microbiology

## Abstract

Engineered metabolic pathways in microbial cell factories often have no natural organization and have challenging flux imbalances, leading to low biocatalytic efficiency. Modular polyketide synthases (PKSs) are multienzyme complexes that synthesize polyketide products via an assembly line thiotemplate mechanism. Here, we develop a strategy named mimic PKS enzyme assembly line (mPKSeal) that assembles key cascade enzymes to enhance biocatalytic efficiency and increase target production by recruiting cascade enzymes tagged with docking domains from type I *cis*-AT PKS. We apply this strategy to the astaxanthin biosynthetic pathway in engineered *Escherichia coli* for multienzyme assembly to increase astaxanthin production by 2.4-fold. The docking pairs, from the same PKSs or those from different *cis*-AT PKSs evidently belonging to distinct classes, are effective enzyme assembly tools for increasing astaxanthin production. This study addresses the challenge of cascade catalytic efficiency and highlights the potential for engineering enzyme assembly.

## Abstract (web summary)

Assembly artificial pathway in design connecting media can increase biosynthetic efficiency, but the choice of connecting media is limited. Here, the authors develop a new protein assembly strategy using a pool of docking peptides from polyketide synthase and show its application in astaxanthin biosynthesis in *E. coli*.

## Introduction

Cell metabolic engineering can enable natural product overproduction under specific culture conditions, which is of interest to academic researchers and industrial engineers in microbial manufacturing. Researchers seek strategies to turn living cells into microbial factories that produce renewable fuels, drugs, and materials^1–3^. Natural biocatalytic systems usually form organized multienzyme complexes, enzyme molecular scaffolds, or reaction microcompartments to facilitate multi-step metabolic reactions by promoting substrate to transfer among the active sites of enzymes clustered in close proximity^4–6^. However, engineered heterologous metabolic pathways in microbial cell factories often have no such organization, resulting in metabolic flux imbalance and low biosynthetic efficiency^7^. Multienzyme assembly can be formed through post-translational self-assembly of enzymes in a sequential biosynthetic cascade mediated by different interactive tools, for example, enzymes directly connect by interacting peptides, or synthetic scaffolds, a separate biomolecule (protein, DNA, RNA) to which the different proteins are docking with specific interaction domains^8–13^—improved metabolic regulation including limiting the diffusion of transient intermediates, facilitating the rapid conversion of toxic intermediates to nontoxic products, preventing crosstalk between competitive metabolic pathways, and preventing the degradation and loss of unstable intermediates^14–16^.

Researchers have consistently investigated multienzyme assembly strategies in vivo and in vitro in the last decade^16,17^. For example, enzymes assembled on a synthetic cellulosome formed by fusing the endoglucanase (CelA) and a cellulose binding domain (CBD) to the C-terminal of orthogonal dCas9 proteins achieved a 2.6-fold and 2.8-fold higher reducing sugar production, respectively, when the multienzyme complex was scaffolded onto a DNA target^13,18^. Multi-domain protein scaffold assembly was performed by fusing a string of protein-binding domains: GTPase binding domain (GBD), Src homology 3 domain (SH3), and PSD95/DlgA/Zo-1 (PDZ) originating from metazoan signaling proteins in various arrangements for fine control of metabolic flux and significant improvement in product synthesis, such as, 77-fold improvement in mevalonate titer^8^, 2-3 folds increase of gamma-aminobutyric acid production^19^, and 29% and 63% increases of violacein and deoxyviolacein production^20^, respectively. The interacting peptides RIDD and RIAD or SpyTag and SpyCatcher are convenient tools in multienzyme assembly with widespread applicability^21–23^. Additional notable examples include the coiled coils possessing intertwined α-helices forming superhelical bundles, affibodies, non-immunoglobulin affinity proteins derived from the Fc-binding domain of *Staphylococcus aureus* protein A, and synthetic carboxysomes (CBs) formed by introducing CB proteins from *Prochlorococcus marinus* MED4 cyanobacteria into *Escherichia coli*^24–26^. However, the limited selection of interactive elements for different application requirements restricts the possibility of multienzyme assembly strategies from reaching their potential.

**Fig. 1:**
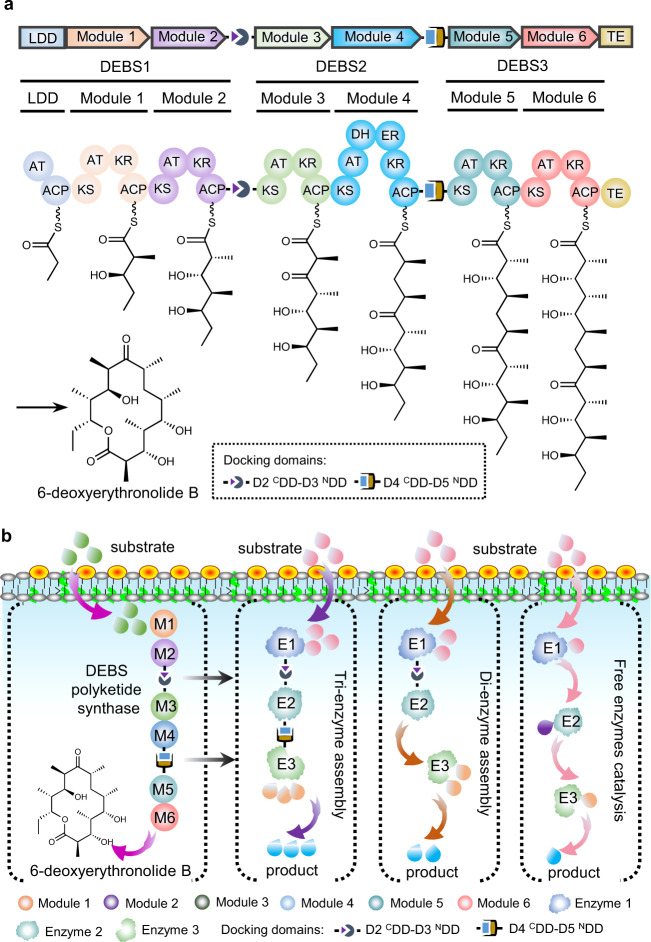
Overview of the mimic PKS enzyme assembly line (mPKSeal) strategy for the assembly of cascade enzymes. **a** Schematic architecture of 6-deoxyerythronolide B synthase (DEBS). DEBS1, −2, and −3 represent the three large subunits connected by docking domains (DDs, D2 ^C^DD-D3 ^N^DD between DEBS1 and DEBS2, and D4 ^C^DD-D5 ^N^DD between DEBS2 and DEBS3). The 6-deoxyerythronolide B is synthesized by the proceeding action of a loading didomain (LDD), six extension modules (M1-M6), and a terminal thioesterase (TE) domain. KS ketosynthase, AT acyltransferase, ACP acyl carrier protein, KR ketoreductase, DH dehydratase, ER enoylreductase, TE thioesterase. **b** The mPKSeal strategy using mDEBSeal to assemble di-enzyme or tri-enzyme units by introducing the DDs (D2 ^C^DD-D3 ^N^DD and/or D4 ^C^DD-D5 ^N^DD) of DEBS. E1: enzyme 1; E2: enzyme 2; E3: enzyme 3.

Type I modular polyketide synthases (PKSs) are assemblies of several enzymes synthesizing diverse polyketide products that are widely used in the clinic, including antibiotics (erythromycin), antiparasitic drugs (avermectin), immunosuppressants (FK506), and cancer chemotherapy (ixabepilone)^27,28^. They are composed of a series of catalytic modules acting successively in polyketide chain elongation, processing, and termination^29^. Typical PKS subunits are tightly homodimeric and contain a few modules each^27,30–32^. Type I modular PKSs can be further classified as *cis*-AT PKS and *trans*-AT PKS. The *cis*-AT PKS modules contain all three essential domains (KS: ketosynthase; AT: acyltransferase; ACP: acyl carrier protein), whereas in the *trans*-AT PKS modules the elongation unit is transacylated onto the ACP domain by a free-standing AT that is often shared across multiple modules^33^. The subunits are considered to associate with each other via interactions. These interactions are mediated, at least in part, by short, independently-folding regions at the extreme C- and N-termini of the subunits, which are referred to as docking domains (DDs)^34^. For example, 6-deoxyerythronolide B synthase (DEBS) is a prototypical modular megasynthase assembling the polyketide core of macrocyclic aglycone of erythromycin, which contains three large subunits (DEBS1, -2, and -3), with each one housing two unique modules (M1 and M2, M3 and M4, or M5 and M6)^27,35–37^ (Fig. 1a). The subunits are connected by short DDs. DEBS1 and DEBS2 interaction DDs (defined as D2 ^C^DD-D3 ^N^DD in this study) are composed of 86 and 31 amino acids, respectively, while DEBS2 and DEBS3 DDs (D4 ^C^DD-D5 ^N^DD in this study) comprise 80 and 39 amino acids, respectively, and form the complete PKS complex of DEBS and facilitate efficient pipeline-like erythromycin biosynthesis^35^ (Fig. 1a). More than three thousand type I modular PKSs with diverse structures containing different DDs have been characterized in nature^27^, such as rapamycin polyketide synthase (RAPS), spinosad polyketide synthase, and salinomycin polyketide synthase containing two^38^, four^39^, and eight^40^ pairs of DDs, respectively. Among these, approximately half of type I modular PKS systems (more than 1600) were annotated as *cis*-AT PKSs^27^, the DDs from which can be further classified into different classes (class 1a, 1b, 2, etc.) which could be orthogonal interaction with each other^34,41–43^. These PKSs contain different numbers of domains and subunits, with the potential interchangeability of the PKSs catalytic modules making them an appealing prospective toolkit for combinatorial biosynthesis of new polyketide products^37,44–47^, rational computational design for new catalytic modules^48^, or to act interactions between fluorescent protein variants^49^.

Astaxanthin is a terpenoid with strong antioxidant properties and is widely used in nutritional supplements and pharmaceuticals, and the worldwide market of astaxanthin is expected to increase dramatically^50^. With the development of metabolic engineering, researchers have tried to engineer microbial producers to produce astaxanthin^51–53^. In previous work, we engineered the biosynthetic pathway of astaxanthin in *E. coli* and obtained a high astaxanthin-producing strain^54^. The engineered biosynthetic pathway included two heterologous pathways with different locations in cell context: the upstream cytosolic mevalonate (MVA) pathway converted acetyl-coenzyme A (Ac-CoA) to the five-carbon molecules isopentenyl pyrophosphate (IPP) and its allylic isomer dimethylallyl pyrophosphate (DMAPP); and the downstream cell membrane localized astaxanthin biosynthetic pathway sequentially condensed IPP and DMAPP to astaxanthin^55^. However, it is difficult to further improve the astaxanthin production via traditional metabolic engineering strategies^51,56^. Therefore, multiple engineering strategies should be developed to break through the astaxanthin-producing bottleneck.

Here, we employ the DDs of type I *cis*-AT PKS as the connecting medium to develop a strategy called mimic PKS enzyme assembly line (mPKSeal) for multienzyme assembly (Fig. 1b). Despite advances toward understanding DDs^57,58^, the DDs excised from the complete PKS used for non-PKS assembly are still poorly studied. The *K*_*D*_ assessment of the purified DDs from DEBS (D2 ^C^DD, D3 ^N^DD, D4 ^C^DD, D5 ^N^DD) show that they retain assembly capacity despite being excised from the native PKS structures. mDEBSeal application in engineered *E. coli* results in the co-localization of the assembling enzymes in vivo and the subsequent increase in astaxanthin production, suggesting that the interaction of the DDs takes place intracellularly and increases biosynthetic efficiency. The capacity of mDEBSeal in the assembly of di-enzymes and tri-enzymes in different cellular contexts (cytoplasmic-, membranous-, and cytoplasmic-membranous enzymes) show that cascade catalysis enzyme assembly increased astaxanthin production. Moreover, the assembly mediated by DDs from other natural PKSs such as RAPS^38^, tacrolimus (FKB) polyketide synthase^59^, and aureothin (AUR) synthase^60^ is also effective in enzyme assembly for increasing astaxanthin production. Furthermore, the DDs pairs from different PKSs which clustered in the same branches of phylogenetic tree belong to the same class show activity in enzyme assembly and increase astaxanthin production. The DDs from type I *cis*-AT PKSs can supply multiple genetic assembly elements, suggesting that the mPKSeal strategy potentially provides a pool of enzyme assembly tools to increase production. This provides an attractive strategy for engineering enzymes with desired characteristics in cellular manufacturing.

## Results

### Interaction of mDEBSeal and mRAPSeal interactive elements in vitro

**Fig. 2:**
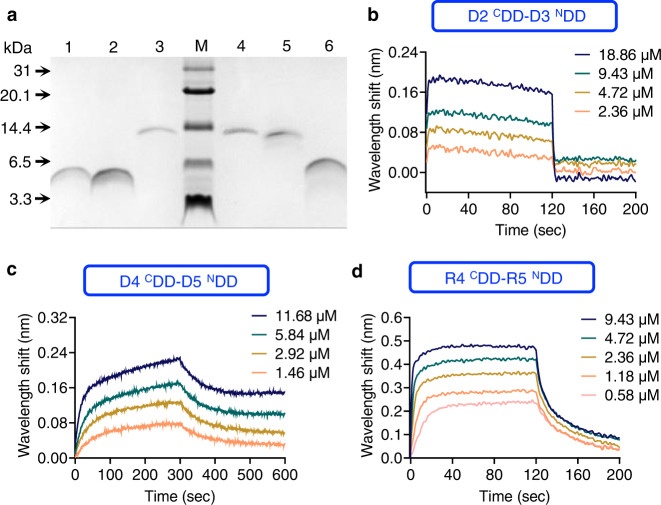
Interaction of DDs. **a** Purified DDs analyzed using 15.5%-Tris-Tricine-SDS-PAGE. Lane 1: D3 ^N^DD-His-tag (5.1 kDa); lane 2: R5 ^N^DD-His-tag (5.1 kDa); lane 3: Flag-tag-R4 ^C^DD (10.6 kDa); lane M: protein standard marker (P1321, Solarbio); lane 4: Flag-tag-D2 ^C^DD (10.6 kDa); lane 5: Flag-tag-D4 ^C^DD (10.7 kDa); lane 6: D5 ^N^DD-His-tag (6.0 kDa). Experiments were repeated at least three times with similar results. **b**–**d** Wavelength shift (nm) generated by the addition of D2 ^C^DD, D4 ^C^DD, or R4 ^C^DD at the concentrations to the complementary D3 ^N^DD, D5 ^N^DD, or R5 ^N^DD, respectively. Source data are provided as a Source Data file.

DEBS (DEBS1, DEBS2, and DEBS3) and RAPS (RapA, RapB, and RapC) are two classical examples each contained two pairs of *cis*-AT PKS DDs^36,38^. The DDs between the subunits comprising approximately 30 or 80 amino acid residues are arranged between two large subunits of DEBS (D2 ^C^DD-D3 ^N^DD between DEBS1 and DEBS2, and D4 ^C^DD-D5 ^N^DD between DEBS2 and DEBS3) and RAPS (R4 ^C^DD-R5 ^N^DD between RapA and RapB, and R10 ^C^DD-R11 ^N^DD between RapB and RapC) and mediate communication between the subunits^35,38^. We suspected that these DDs could be used to recruit engineered enzymes for clustering. The interactions of the DD pairs D2 ^C^DD and D3 ^N^DD, D4 ^C^DD and D5 ^N^DD, and R4 ^C^DD and R5 ^N^DD—excised from the original multienzyme subunits of PKS, overexpressed in *E. coli* Rosetta(DE3)^61^, and purified—were tested in vitro, to evaluate their affinities. Maltose binding protein (MBP) was fused with the DDs for enhanced overexpression and simultaneously fused with His-tag or Flag-tag for Ni^2+^-affinity chromatography or anti-flag affinity chromatography purification. The MBP tag was cleaved by TEV protease and separated from the DDs using Ni^2+^-affinity chromatography, anti-flag affinity chromatography, or size exclusion chromatography (SEC). Ni^2+^-affinity chromatography and SEC were performed for the N-terminal DDs (^N^DDs) purification. Ni^2+^-affinity chromatography and anti-flag affinity chromatography were performed for C-terminal DD (^C^DDs) purification because of the close elution time between MBP and ^C^DDs in SEC. The DDs were detected using Tris-Tricine-SDS PAGE^62^ (Fig. 2a).

Kinetic analysis of the purified DDs was performed using Bio-Layer Interferometry (BLI; a label-free technology for measuring biomolecular interactions)^63^. The ~10 kDa ^C^DDs (D2 ^C^DD, D4 ^C^DD, or R4 ^C^DD) were free in the working solution, while the ~5 kDa ^N^DDs (D3 ^N^DD, D5 ^N^DD, or R5 ^N^DD) were immobilized onto the biosensor tip. The binding of ^C^DDs in solution to the immobilized ^N^DDs resulted in an increase in optical thickness, which caused a shift in wavelengths monitored by the detector. Monitoring the interference pattern in real time provided kinetic binding data of the D2 ^C^DD-D3 ^N^DD, D4 ^C^DD-D5 ^N^DD, and R4 ^C^DD-R5 ^N^DD (Fig. 2b-d). The *K*_*D*_ value of D2 ^C^DD-D3 ^N^DD (14.6±4.7 μM) and D4 ^C^DD-D5 ^N^DD (0.28±0.08 μM) showed close values compared with the reported interactions of the native biocatalytic modules [1.1 μM (D2 ^C^DD-D3 ^N^DD) and 2.1 μM (D4 ^C^DD-D5 ^N^DD)^58^], indicating that the interaction activities of the excised DDs from the native multienzyme complex retained their assembly properties. R4 ^C^DD-R5 ^N^DD showed interaction with *K*_*D*_ = 0.34±0.03 μM which has not been reported. Binding assays were performed three times. In contrast, no interaction was detected between D2 ^C^DD-D5 ^N^DD and R4 ^C^DD-D3 ^N^DD (Supplementary Fig. 1), demonstrating the intrinsic incompatibility of the DD pairs. Above all, the excised DDs could be used to assemble or relocate heterologous enzymes, instead of biocatalytic subunits of native PKS, to form an artificial multienzyme complex in vitro.

### mDEBSeal and mRAPSeal construction for multienzyme assembly in vivo

**Fig. 3:**
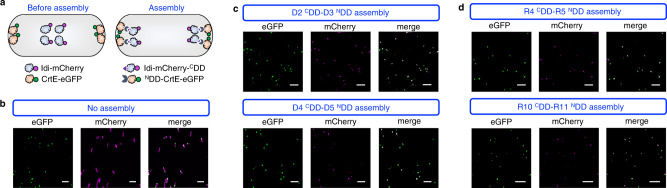
Formation of mDEBSeal and mRAPSeal multienzyme assembly following the interaction of DDs in *E. coli*. **a** Co-localization of Idi-mCherry tagged with ^C^DDs (Idi-mCherry-^C^DD) and CrtE-eGFP tagged with ^N^DDs (^N^DD-CrtE-eGFP). Idi-mCherry and CrtE-eGFP without tagged DD were used as the negative control. Blue filled cloud: Idi; orange filled cloud: CrtE; violet filled circle: mCherry; green filled circle: eGFP; violet filled triangle: ^C^DD; dark blue filled incomplete circle: ^N^DD. **b** Confocal microscopy images of cells expressing unassembled Idi-mCherry and CrtE-eGFP. **c** Confocal microscopy images of cells expressing Idi-mCherry and CrtE-eGFP tagged with D2 ^C^DD-D3 ^N^DD and D4 ^C^DD-D5 ^N^DD from DEBS, respectively. **d** Confocal microscopy images of cells expressing Idi-mCherry and CrtE-eGFP tagged with R4 ^C^DD-R5 ^N^DD and R10 ^C^DD-R11 ^N^DD from RAPS, respectively. **b**–**d** Scale bar: 5 μm. Experiments were repeated at least three times with similar results.

The assembly capacity of DDs for retaining Idi and CrtE enzyme activity in vivo was investigated. Idi is dispersed in the cytoplasm, while CrtE is located in the poles of the engineered *E. coli* astaxanthin-producing A0 strain^21^. DDs with fluorescent proteins were tagged to the N terminal and/or C terminal of Idi and CrtE, respectively, to detect enzyme assembly by fluorescence co-localization (Fig. 3a). The red fluorescent protein (mCherry) and enhanced green fluorescent protein (eGFP) were fused to the C terminal of Idi (Idi-mCherry) and CrtE (CrtE-eGFP). The D2 ^C^DD and D4 ^C^DD of DEBS were tagged to the C terminal of Idi-mCherry to form Idi-mCherry-D2 ^C^DD and Idi-mCherry-D4 ^C^DD. D3 ^N^DD and D5 ^N^DD were tagged to the N terminal of CrtE-eGFP to form D3 ^N^DD-CrtE-eGFP and D5 ^N^DD-CrtE-eGFP. Plasmids Idi-mCherry-^C^DD and ^N^DD-CrtE-eGFP were co-transformed into *E. coli* BL21(DE3) to visualize their locations using confocal microscopy. The strain containing Idi-mCherry and CrtE-eGFP without DDs was used as the control. mCherry and eGFP without tagged DDs individually dispersed through the cytosol and near the pole of cells, respectively (Fig. 3b). mCherry and eGFP with adaptive D2 ^C^DD-D3 ^N^DD and D4 ^C^DD-D5 ^N^DD co-localized near the poles of the cells (Fig. 3c).

The two co-localized fluorescence proteins of the RAPS DD pairs R4 ^C^DD-R5 ^N^DD and R10 ^C^DD-R11 ^N^DD showed their DDs assembly capacity (Fig. 3d). These results indicated that the pairs of excised DDs of DEBS and RAPS retain multienzyme assembly in vivo. No fluorescent co-localization occurred using the undocking pairs of D2 ^C^DD-D5 ^N^DD, D4 ^C^DD-D3 ^N^DD, R4 ^C^DD-R11 ^N^DD, and R10 ^C^DD-R5 ^N^DD from the same PKS (Supplementary Fig. 2). It indicated that the heterologous enzymes assembly mediated by the excised DDs could sequentially assemble in vivo, which is consistent with the native PKS^58,64^.

### Utilizing mDEBSeal in di-enzyme and tri-enzyme assemblies

**Fig. 4:**
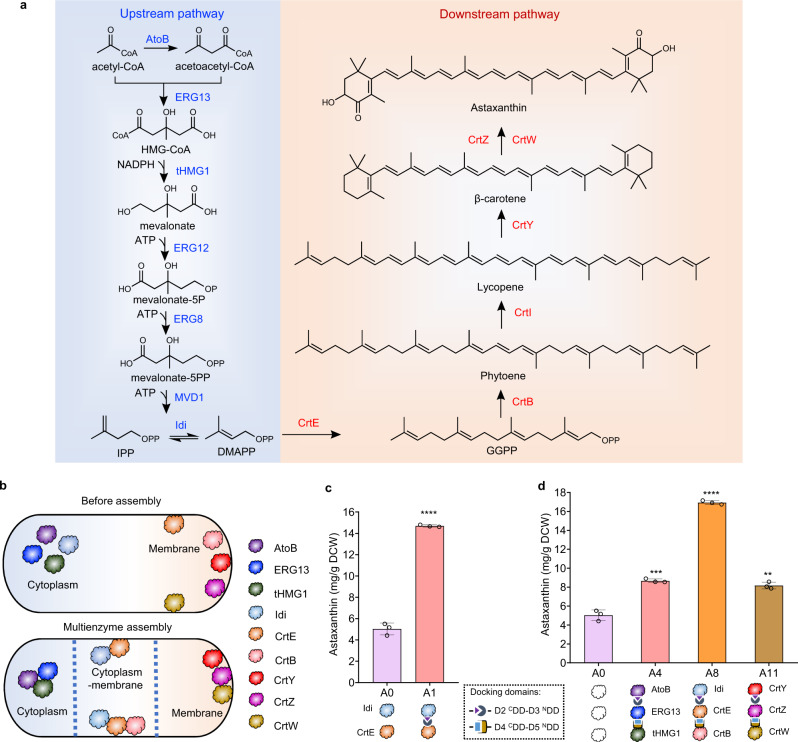
mDEBSeal strategy to improve the cascade biocatalytic efficiency of the astaxanthin biosynthetic pathway. **a** The engineered biosynthetic pathway of astaxanthin. The enzymes of the upstream mevalonate (MVA) pathway (blue) are dispersed in the cytosol, whereas enzymes in the downstream pathway (red) are located at the cell membrane. CoA, coenzyme A; HMG-CoA, (S)−3-hydroxy-3-methylglutaryl-CoA; mevalonate-5P, mevalonate-5-phosphate; mevalonate-5PP, mevalonate diphosphate; IPP, isopentenyl diphosphate; DMAPP, dimethylallyl diphosphate; GGPP, geranylgeranyl diphosphate. AtoB, acetyl-CoA C-acetyltransferase; ERG13, 3-hydroxy-3-methylglutaryl CoA synthase; tHMG1, 3-hydroxy-3-methylglutaryl CoA reductase; ERG12, mevalonate kinase; ERG8, phosphomevalonate kinase; MVD1, mevalonate diphosphate decarboxylase; Idi, isopentenyl diphosphate isomerase; CrtE, geranylgeranyl diphosphate synthase; CrtB, phytoene synthase; CrtI, phytoene desaturase; CrtY, lycopene β-cyclase; CrtZ, β-carotene hydroxylase; CrtW, β-carotene ketolase. **b** mDEBSeal di-enzyme and tri-enzyme assembly of the astaxanthin biosynthetic pathway based on the cellular localization of enzymes (cytoplasmic-, cytoplasmic-membranous, and membranous). Purple filled cloud: AtoB; dark blue filled cloud: ERG13; green filled cloud: tHMG1; blue filled cloud: Idi; orange filled cloud: CrtE;  pink filled cloud: CrtB; red filled cloud: CrtY; violet filled cloud: CrtZ; ginger filled cloud: CrtW. **c** mDEBSeal di-enzyme (Idi-CrtE) assembly to improve astaxanthin production. Strain A1 contained D2 ^C^DD-D3 ^N^DD tagged to Idi and CrtE, respectively. Strain A0 without DDs acted as the control. **d** mDEBSeal tri-enzyme assembly to improve astaxanthin production. Strain A4 contained the cytoplasmic tri-enzyme assembly of AtoB-ERG13-tHMG1 tagged with D2 ^C^DD-D3 ^N^DD and D4 ^C^DD-D5 ^N^DD. Strain A8 contained the cytoplasmic-membranous tri-enzyme assembly of Idi-CrtE-CrtB tagged with D2 ^C^DD-D3 ^N^DD and D4 ^C^DD-D5 ^N^DD. Strain A11 contained a membranous tri-enzyme assembly of CrtY-CrtZ-CrtW tagged with D2 ^C^DD-D3 ^N^DD and D4 ^C^DD-D5 ^N^DD. Strain A0 without DDs was used as the control. Empty filled cloud: candidate assembly enzyme. **c**, **d** Experiments were repeated three times, and each point represents the mean ± SD. Statistical analysis was performed using a two-tailed Student’s *t*-test (****P < 0.0001, ***P < 0.001, **P < 0.01). *P* value in **c**, *P*^*A1*^ < 0.0001. *P* values in **d**, *P*^*A4*^ = 0.0004, *P*^*A8*^ < 0.0001, *P*^*A11*^ = 0.0012. Source data are provided as a Source Data file.

The production of complex bio-based chemicals for applications such as the rapid conversion of toxic intermediates to products using multienzyme complex is increasing^5,6,16^. The mDEBSeal was probed for potential improvements in biocatalytic efficiency by examining the assembly of biosynthetic pathway enzymes involved in astaxanthin production. In previous work, we engineered the biosynthetic pathway of astaxanthin in *E. coli* (Fig. 4a) and obtained a high-astaxanthin-producing strain (labeled as A0 in this study)^54^ which showed intermediates accumulation of Ac-CoA, acetoacetyl-CoA (Ac-Ac-CoA), IPP/DMAPP, and β-carotene. Here, we attempted to reduce accumulation of the detected intermediates by facilitated assembly of the relevant enzymes. On the other hand, the differential location of the enzymes in the engineered pathway enabled us to explore the assembly potentials of mDEBSeal for enzymes located in different spaces and assess improvements in astaxanthin production compared with strain A0.

The supply of IPP and DMAPP poses a bottleneck to downstream astaxanthin biosynthesis; therefore, increasing the supply of these C5 building blocks is beneficial for improving astaxanthin production^65^. However, excessive IPP and DMAPP accumulation is cytotoxic^66^. This dilemma was resolved in our previous study by employing a modular enzyme assembly strategy using relatively RIDD and RIAD peptide pairs for the construction of metabolite nodes (Idi and CrtE) to streamline the flux of carotenoid biosynthesis and improve product titers^21^. Therefore, Idi-CrtE were selected as the target enzymes to carry out di-enzyme assembly by mDEBSeal in *E. coli* (Fig. 4b). The D2 ^C^DD and D3 ^N^DD were tagged to the C- and N-terminals of Idi and CrtE, respectively, resulting in strain A1 containing Idi-D2 ^C^DD and D3 ^N^DD-CrtE. A1 produced 14.7 mg/g dry cell weight (DCW) of astaxanthin, which was 1.79-fold higher than that produced by strain A0 (5.0 mg/g DCW) (Fig. 4c). The strains containing only one of the DDs were also compared, strain A2 (containing only Idi-D2 ^C^DD) produced the same amount of astaxanthin as strain A0, while strain A3 (containing D3 ^N^DD-CrtE, 7.5 mg/g DCW) produced more astaxanthin compared with strain A0 (Supplementary Fig. 3a). However, astaxanthin production in assembly strain was still higher than that in control strains, suggesting that the strategy assembling Idi and CrtE improved the target biosynthetic efficiency.

Considered the high accumulation of Ac-CoA and Ac-Ac-CoA in strain A0, as well as the reported that the assembly of AtoB-ERG13-tHMG1 obtained dramatically increase of mevalonate^8^, a tri-enzyme assembly composed of cytosolic AtoB, ERG13 and tHMG1 enzymes of the MVA pathway^55^ was performed using the mDEBSeal to streamline astaxanthin production. The D2 ^C^DD and D3 ^N^DD were tagged to the C-terminal of AtoB and N-terminal of ERG13, and the D4 ^C^DD and D5 ^N^DD were tagged to the C-terminal of ERG13 and N-terminal of tHMG1 to form strain A4 containing AtoB-D2 ^C^DD, D3 ^N^DD-ERG13-D4 ^C^DD, and D5 ^N^DD-tHMG1. Strain A4 produced 8.7 mg/g DCW of astaxanthin, which was 72.4% higher than strain A0 (Fig. 4d). This suggested that applying mDEBSeal to the assembly of cytoplasmic tri-enzymes improved the concentration of final product.

Cytoplasmic Idi and membrane-bound CrtE are the key nodes for astaxanthin biosynthesis, as they connect the upstream and downstream pathways^21^. Sequential catalysis was applied in the assembly of Idi, CrtE, and another membrane-bound enzyme (CrtB) using the mDEBSeal strategy by tagging the DDs to produce strain A8 containing Idi-D2 ^C^DD, D3 ^N^DD-CrtE-D4 ^C^DD, and D5 ^N^DD-CrtB. This strain produced 16.9 mg/g DCW of astaxanthin which is a 2.4-fold increase in astaxanthin production compared with strain A0 (Fig. 4d).

Membrane-bound CrtZ and CrtW are key steps in astaxanthin synthesis because of the alternative reactions and numerous intermediates^67^. A tri-enzyme assembly composed of membrane-bound CrtY, CrtZ, and CrtW was performed. Strain A11 was produced harboring CrtY-D2 ^C^DD, D3 ^N^DD-CrtZ-D4 ^C^DD, and D5 ^N^DD-CrtW, which produced 8.2 mg/g DCW of astaxanthin, which was consistent with the expectation that the DDs engaged in forming a membrane-bound tri-enzyme assembly to improve astaxanthin biosynthesis. The higher astaxanthin production in strain A8 compared with A4 and A11 indicated that the cytoplasmic-membranous tri-enzyme assembly of mDEBSeal was the optimized model for enzyme assembly in this engineered astaxanthin biosynthetic pathway.

A comparison of the control strains containing only one or two of the DDs compared with the start strain A0 showed that the DDs themselves had no effect on the astaxanthin production of strains, with the exception of strain A13 containing D3 ^N^DD-CrtZ-D4 ^C^DD (1.0 mg/g DCW), which produced less astaxanthin, and strain A9 containing D3 ^N^DD-CrtE-D4 ^C^DD (11. 4 mg/g DCW), which produced more astaxanthin (Supplementary Fig. 3b–d). Therefore, there might be some unpredictable variability when tagging some specific membrane-bound enzymes using the mDEBSeal for multienzyme assembly.

Comparing the accumulation of the carotenoids in strains A0, A1, A4, A8, and A11 showed that the production of astaxanthin (final product) was higher in all strains that employed the multienzyme assembly strategy than that in the control A0. Interestingly, not all strains had higher total carotenoids content than that in the control A0 (Supplementary Fig. 4). The assembly of cytoplasmic Idi and membrane-bound CrtE can increase the accumulation of the final product astaxanthin as well as the total carotenoids. This result showed that the flux of the target pathway was opened and the accumulation of both intermediates and total carotenoids increased substantially, with the metabolism of Idi and CrtE being a key node in enhancing the overall synthetic efficiency. The assembly of the upstream cytoplasmic enzymes AtoB, ERG13, and tHMG1 can increase the accumulation of the final product astaxanthin, but decreases the intermediate carotenoids, while the total carotenoids did not change. This engineering of upstream pathway seems not to increase the whole flux but the final product accumulation. Similar to the results of Idi-CrtE, which opened up the flux of the target pathway, the assembly of cytoplasmic Idi and membrane-bound CrtEB can increase the accumulation of the final product astaxanthin and the total carotenoids. However, both Idi-CrtE and Idi-CrtEB had the same accumulation of total carotenoids, but different ratios of the different intermediates. This implied that assembly of the key node enzymes can enhance the overall synthetic efficiency, while assembly of the non-key node enzymes can regulate the intermediates accumulation. The assembly of membrane-bound CrtYZW can increase the accumulation of final product astaxanthin, but decreased the total carotenoids and intermediates. This result showed that this assembly was in favor of a transformation of the intermediate carotenoids to astaxanthin. The assembly strategy allows to assemble selected catalytic enzymes (key node of the target pathway) to increase the total metabolic flux or to transform a certain intermediate to a target product (non-key node of the target pathway).

### Expanding mPKSeal using the DDs from natural type I *cis*-AT PKSs

**Fig. 5:**
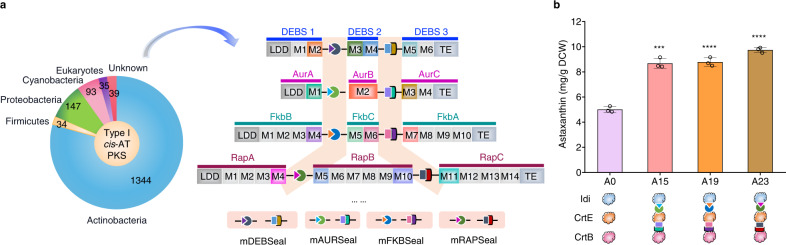
Enzyme assembly mediated by DDs from natural type I *cis*-AT PKSs. **a** Distribution of type I *cis*-AT PKSs among the different phyla. More than 1600 type I *cis*-AT PKSs are characterized in nature containing different pairs of DDs. DEBS, aureothin synthase (AURS), tacrolimus polyketide synthase (FKBS), and rapamycin polyketide synthase (RAPS) from the Actinobacteria all include two pairs of DDs: D2 ^C^DD-D3 ^N^DD and D4 ^C^DD-D5 ^N^DD, A1 ^C^DD-A2 ^N^DD and A2 ^C^DD-A3 ^N^DD, F4 ^C^DD-F5 ^N^DD and F6 ^C^DD-F7 ^N^DD, and R4 ^C^DD-R5 ^N^DD and R10 ^C^DD-R11 ^N^DD, respectively. LDD, loading didomain; M1-M14, extension modules; TE, thioesterase domain. **b** Tri-enzyme assembly increased astaxanthin production in shake-flask fermentation. mAURSeal strategy was carried out in strain A15 containing the Idi-CrtE-CrtB assembly tagged with A1 ^C^DD-A2 ^N^DD and A2 ^C^DD-A3 ^N^DD from AURS. mFKBSeal strategy was carried out in strain A19 containing the Idi-CrtE-CrtB assembly tagged with F4 ^C^DD-F5 ^N^DD and F6 ^C^DD-F7 ^N^DD from FKBS. mRAPSeal strategy was carried out in strain A23 containing the Idi-CrtE-CrtB assembly tagged with R4 ^C^DD-R5 ^N^DD and R10 ^C^DD-R11 ^N^DD from RAPS. Strain A0 without DDs was used as the control. Experiments were repeated three times, and each point represents the mean ± SD. Statistical analysis was performed using a two-tailed Student’s *t*-test (****P < 0.0001, ***P < 0.001). *P*^*A15*^ = 0.0002, *P*^*A19*^ = 0.0001, *P*^*A23*^ < 0.0001. Source data are provided as a Source Data file.

Type I *cis*-AT PKSs are diverse in nature with over 1600 currently characterized, and the majority are obtained from bacterial sources^27^ (Fig. 5a). The diverse *cis*-AT PKSs systems comprise numbers of DDs, such as DDs of AURS (from *Streptomyces thioluteus*^60^), FKBS (from *Streptomyces* sp. MA6548^59^), and RAPS (from *Streptomyces rapamycinicus*^38^)(Fig. 5a). The most efficient cytoplasmic-membranous tri-enzyme assembly model of Idi-CrtE-CrtB was recruited to investigate the assembly of the DDs from different natural PKSs. Strain A15 containing Idi-A1 ^C^DD, A2 ^N^DD-CrtE-A2 ^C^DD, and A3 ^N^DD-CrtB; strain A19 containing Idi-F4 ^C^DD, F5 ^N^DD-CrtE-F6 ^C^DD, and F7 ^N^DD-CrtB; and strain A23 containing Idi-R4 ^C^DD, R5 ^N^DD-CrtE-R10 ^C^DD, and R11 ^N^DD-CrtB were obtained using the same construction strategy by replacing the different DDs. A15 (8.7 mg/g DCW), A19 (8.8 mg/g DCW), and A23 (9.7 mg/g DCW) showed increased astaxanthin production compared with strain A0 (Fig. 5b).

Astaxanthin production in control strains containing only one or two of the DDs, such as strain A17 (A2 ^N^DD-CrtE-A2 ^C^DD, 5.2 mg/g DCW), A21 (F5 ^N^DD-CrtE-F6 ^C^DD, 5.0 mg/g DCW), and A25 (R5 ^N^DD-CrtE-R10 ^C^DD, 4.7 mg/g DCW), were comparable with A0 (Supplementary Fig. 5). This indicated that few DDs might be similar with that from DEBS and affect enzyme activity and suggested that DDs from different natural *cis*-AT PKSs can also participate in mPKSeal multienzyme assembly. This potentially enables the usage of DDs from the natural *cis*-AT PKSs containing at least two pairs of DDs as assembly tools to provide numerous of options to streamline target biosynthetic pathways and improve biosynthetic efficiency.

### Expanding mPKSeal by classifying DDs from different type I *cis*-AT PKSs

Engineering the type I modular PKS assembly line for recombinant production has become one of the most promising ways to produce diverse polyketide analogues with potent activities in recent years^68^ by combining subunits joined with DDs from different PKS systems^44,69^. However, not all recombinant PKSs could be assembled and possess biosynthetic capacity. To understand the rules that governed interactions between these DDs, researchers constructed a predictive code to determine the specificity of PKS subunit interactions by a small number of residues in the C-terminus (head, H) and N-terminus (tail, T)^34,41,70^. Phylogenetic clustering analysis based on co-evolution protein-protein interaction corresponding to structural classification suggested that there are mainly three mutually incompatible classes (*H1a–T1a*, *H1b–T1b*, and *H2–T2*), and a single PKS complex might contain the DDs from multiple classes^34,41,43,70^. Therefore, the DDs applied in mPKSeal could be further classified. The D2 ^C^DD-D3 ^N^DD and D4 ^C^DD-D5 ^N^DD pairs of DEBS belong to *H1a–T1a* (*H1a*_*D2 CDD*_ *–T1a*_*D3 NDD*_ and *H1a*_*D4 CDD*_*–T1a*_*D5 NDD*_). The R4 ^C^DD-R5 ^N^DD and R10 ^C^DD-R11 ^N^DD pairs of RAPS or F4 ^C^DD-F5 ^N^DD and F6 ^C^DD-F7 ^N^DD pairs of FKBS belong to *H1b–T1b* and *H1a–T1a*, respectively (*H1b*_*R4 CDD*_*–T1b*_*R5 NDD*_ or *H1b*_*F4 CDD*_*–T1b*_*F5 NDD*_ and *H1a*_*R10 CDD*_*–T1a*_*R11 NDD*_ or *H1a*_*F6 CDD*_*–T1a*_*F7 NDD*_, respectively).

**Fig. 6:**
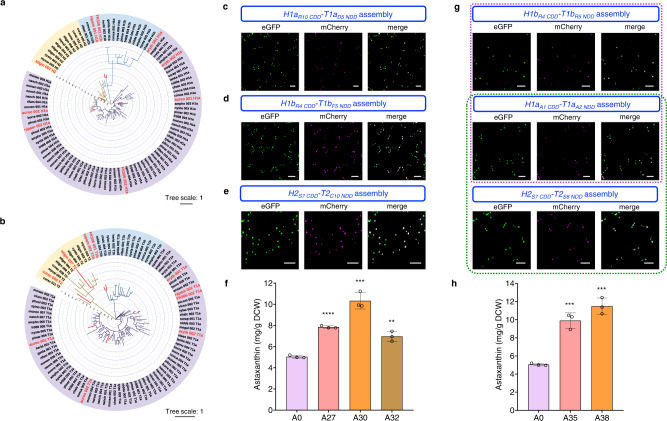
Enzyme assembly mediated by classified DDs from different type I *cis*-AT PKSs. **a** Phylogenetic analysis of ^C^DDs (head, H). Purple: *H1a* class; blue: *H1b* class; yellow: *H2* class; red: classified “H” DDs selected. **b** Phylogenetic analysis of ^N^DDs (tail, T). Purple: *T1a* class; blue: *T1b* class; yellow: *T2* class; red: classified “T” DDs selected. The samples were labeled by the PKS pathway it belongs to, and the interface number within that pathway. **c**–**e** Confocal microscopy images of cells expressing Idi-mCherry and CrtE-eGFP tagged with *H1a*_*R10 CDD*_ and *T1a*_*D5 NDD*_, *H1b*_*R4 CDD*_ and *T1b*_*F5 NDD*_, or *H2*_*S7 CDD*_ and *T2*_*C10 NDD*_, respectively. **f** Astaxanthin production of strains containing one pair of combinatorial DDs. Strain A27, A30, and A32 contained the di-enzyme assembly of Idi-CrtE tagged with *H1a*_*R10 CDD*_-*T1a*_*D5 NDD*_, *H1b*_*R4 CDD*_-*T1b*_*F5 NDD*_, or *H2*_*S7 CDD*_-*T2*_*C10 NDD*_, respectively. Strain A0 without DDs was used as the control. **g** Confocal microscopy images of cells expressing Idi-mCherry and CrtE-eGFP tagged with *H1a*_*A1 CDD*_ and *T1a*_*A2 NDD*_, *H1b*_*R4 CDD*_ and *T1b*_*R5 NDD*_, or *H2*_*S7 CDD*_ and *T2*_*S8 NDD*_. Purple and green dotted box: combinatorial usage of *H1a*_*A1 CDD*_ and *T1a*_*A2 NDD*_ and *H1b*_*R4 CDD*_ and *T1b*_*R5 NDD*_, and *H1a*_*A1 CDD*_ and *T1a*_*A2 NDD*_ and *H2*_*S7 CDD*_ and *T2*_*S8 NDD*_ in one system. **h** Astaxanthin production by combinatorial usage of two pairs of DDs from different PKS systems. Strain A35 contained the tri-enzyme assembly of Idi-CrtE-CrtB tagged with *H1a*_*A1 CDD*_ and *T1a*_*A2 NDD*_ and *H1b*_*R4 CDD*_ and *T1b*_*R5 NDD*_. Strain A38 contained the tri-enzyme assembly of Idi-CrtE-CrtB tagged with *H1a*_*A1 CDD*_ and *T1a*_*A2 NDD*_ and *H2*_*S7 CDD*_ and *T2*_*S8 NDD*_. Strain A0 without DDs was used as the control. **c**–**e**, **g** Scale bar: 5 μm. Experiments were repeated at least three times with similar results. **f**, **h** Experiments were repeated three times, and each point represents the mean ± SD. Statistical analysis was performed using a two-tailed Student’s *t* test (****P < 0.0001, ***P < 0.001, **P < 0.01). *P* values in **f**, *P*^*A27*^ < 0.0001, *P*^*A30*^ = 0.0003, *P*^*A32*^ = 0.0024. *P* values in **h**, *P*^*A35*^ = 0.0006, *P*^*A38*^ = 0.0003. Source data are provided as a Source Data file.

The classified DDs for di-enzyme assembly were evaluated to explore whether the DDs belonging to the same class derived from different PKSs could be used in one mPKSeal system. The DDs pairs of *H1a*_*R10 CDD*_*–T1a*_*D5 NDD*_, *H1b*_*R4 CDD*_*–T1b*_*F5 NDD*_, and *H2*_*S7 CDD*_*–T2*_*C10 NDD*_ were selected based on the evolutionary tree (Fig. 6a, b). The candidate DDs were fused with Idi-mCherry and CrtE-eGFP at their C- or N-termini accordingly. The co-localization of mCherry and eGFP indicated that the *H1a*_*R10 CDD*_*–T1a*_*D5 NDD*_, *H1b*_*R4 CDD*_*–T1b*_*F5 NDD*_, and *H2*_*S7 CDD*_*–T2*_*C10 NDD*_ DDs from different PKSs, but the the same class clustered in the same branches of phylogenetic tree, were assembled (Fig. 6c-e). Astaxanthin production of strain A27 (Idi-*H1a*_*R10 CDD*_ and *T1a*_*D5 NDD*_-CrtE; 7.9 mg/g DCW), strain A30 (Idi-*H1b*_*R4 CDD*_ and *T1b*_*F5 NDD*_-CrtE; 10.3 mg/g DCW), and strain A32 (Idi-*H2*_*S7 CDD*_ and *T2*_*C10 NDD*_-CrtE; 7.0 mg/g DCW) were higher compared with that of strain A0 (Fig. 6f). Moreover, astaxanthin production of strains containing only one DD presented similar values compared with strain A0 (Supplementary Fig. 6a).

We further evaluated the classified DDs for tri-enzyme assembly. The two DDs pairs from the same PKS belonging to the same classes (*H1a*_*D2 CDD*_ *–T1a*_*D3 NDD*_ and *H1a*_*D4 CDD*_*–T1a*_*D5 NDD*_ from DBES), or different classes (*H1b*_*R4 CDD*_*–T1b*_*R5 NDD*_ and *H1a*_*R10 CDD*_*–T1a*_*R11 NDD*_ from RAPS) could be used in combination for Idi-CrtE-CrtB tri-enzyme assembly to improve astaxanthin production (Fig. 5b). Two DD pairs (*H1a*_*A1 CDD*_*–T1a*_*A2 NDD*_ and *H1b*_*R4 CDD*_*–T1b*_*R5 NDD*_, and *H1a*_*A1 CDD*_*–T1a*_*A2 NDD*_ and *H2*_*S7 CDD*_*-T2*_*S8 NDD*_) tagged to Idi-mCherry and CrtE-eGFP, respectively, were employed to determine whether the DDs belonging to the same class derived from different PKSs could assemble enzymes in one system. A1 ^C^DD-A2 ^N^DD (*H1a-T1a*) was from AURS, R4 ^C^DD-R5 ^N^DD belonged to *H1b–T1b*, and S7 ^C^DD-S8 ^N^DD of stigmatellin synthase from *Stigmatella aurantiaca*^71^ belonged to *H2–T2*. Fluorescent co-localization showed that *H1a*_*A1 CDD*_*–T1a*_*A2 NDD*_, *H1b*_*R4 CDD*_*–T1b*_*R5 NDD*_, and *H2*_*S7 CDD*_*-T2*_*S8 NDD*_ were assembled (Fig. 6g), while *H1a*_*A1 CDD*_*–T1b*_*R5 NDD*_, *H1b*_*R4 CDD*_*–T1a*_*A2 NDD*_, *H1a*_*A1 CDD*_*–T2*_*S8 NDD*_, and *H2*_*S7 CDD*_*–T1a*_*A2 NDD*_ were not (Supplementary Fig. 7). Astaxanthin production levels of strain A35 harboring *H1a*_*A1 CDD*_*–T1a*_*A2 NDD*_ and *H1b*_*R4 CDD*_*–T1b*_*R5 NDD*_ (9.9 mg/g DCW) and strain A38 harboring *H1a*_*A1 CDD*_*–T1a*_*A2 NDD*_ and *H2*_*S7 CDD*_*-T2*_*S8 NDD*_ (11.5 mg/g DCW) increased compared with A0 (Fig. 6h). The control strains containing one or two DDs showed no significant differences in astaxanthin production compared with strain A0 (Supplementary Fig. 6b).

## Discussion

Over the last two decades, diverse approaches were explored to generate new polyketide compounds by engineering PKSs^68^, with many focused on swapping and replacing PKS modules and functional domains, or modifying the active sites to induce higher substrate promiscuity or produce novel polyketides^37,44–47^. However, there is a lack of in-depth understanding for suitably applying the DDs isolated from PKSs. This study determined that the DDs from type I *cis*-AT PKS can be used to mediate assembly between discrete pathway enzymes to improve the target production. Our findings of the applying of mPKSeal for multienzyme assembly by introducing the DDs to co-localize the fluorescence fusions and to improve biocatalytic efficiency provided solid evidence for the assembly capacity of the DDs in engineered cell factories (Figs. 3–5). This expanded the applications of the elements from PKS systems, and generated more possibilities for the engineering of multienzyme assembly. This study makes a contribution to the literature by employing the DDs of type I *cis*-AT PKS to the multienzyme assembly and developed a synthetic library of enzyme assembly elements.

The DDs usually range from twenty to dozens of amino acids enabling us to manipulate them easily, meanwhile, they are modular elements which do not interact with adjacent proteins, and which retain their functions when relocated. This inspired us to use these DDs as mediated interfaces to construct artificial multienzyme complexes by directed co-localization of cascade enzymes catalyzing sequential biochemical reactions for target production. The mPKSeal strategy was used to assemble di-enzymes and tri-enzymes in different cellular contexts and improved the biocatalytic efficiency of metabolic pathway enzymes in different spatial locations to increase astaxanthin accumulation. The assembly of the key node enzymes can enhance the overall synthetic efficiency, but the assembly of the non-key node enzymes can regulate the intermediates accumulation. It is worth mentioning that the starting strain for astaxanthin production used in this study (A0) is an optimized high-producing strain^54^, and it is difficult to further improve the astaxanthin production via metabolic engineering^51,56^. However, compared with the multi-steps engineering to increase the astaxanthin production by the metabolic engineering strategies (*e.g*., engineering promoters, tuning gene copy numbers, balancing co-factors and energy supply^56^, etc.), a significant increase (2.4-fold, and up to 16.9 mg/g DCW) in astaxanthin production was obtained by simply engineering involving key enzyme assembly through the introduction of DDs compared with A0. This mPKSeal strategy displayed simplicity, convenience, and high product formation efficiency. Moreover, this study showed that the performance of most assembly enzymes tagged with DDs were unaffected, despite unpredictable effects observed for some membrane enzymes. For example, CrtE tagged with D3 ^N^DD and D4 ^C^DD increased the target production (Supplementary Fig. 3a, c), whereas CrtZ tagged with D3 ^N^DD and D4 ^C^DD decreased production (Supplementary Fig. 3d). It appears that certain excess DD affect the catalytic efficiency of some membrane proteins, which should be paid attention in the application of mPKSeal. Regardless, the mPKSeal strategy presents powerful application potential to improve cascade biocatalytic reaction efficiency. Although two sequential enzymes can also be physically linked together through direct fusion, we have shown that the enzyme assembly strategy produced complexes with higher catalytic efficiency than enzyme fusions in *E. coli* cells (Supplementary Fig. 8). Compared with RIAD-RIDD assembly strategy which could support assembly at 1:2 ratio, mPKSeal was conducted at equivalent stoichiometry. In assembling the same key node of the pathway for carotenoids production in the strain A0, RIAD-RIDD had better benefit than mPKSeal, which implied that considering the proportion of enzyme assembly can optimize the assembly effect in some cases (Supplementary Fig. 4). This study provides a means to manipulate the physical location of enzymes without changing the abundance and holds potential to produce high-yield strains. To compensate for the difference in kinetic parameters of the target enzymes, the number of enzymes can be balanced by adjusting the ratio of the target enzymes by tandem DDs to improve the production of the target product.

The limited number of existing interactive elements restricts the broad application of multienzyme assembly strategy. In this study, mPKSeal strategy supported a big potential library. The DDs used in this study for di- and tri-enzyme assembly provided a proof-of-concept showing that type I *cis*-AT PKSs containing different pairs of DDs (2, 3, 4, 5, or more) characterized in nature^27^ could be a pool of docking tools for particular enzyme assembly. Phylogenetic clustering analysis based on co-evolution detection algorithm showed that these DDs can be classified into different classes^41^. These DDs could be used in combination in one system and enable a greater selection of DDs from different PKSs for multienzyme assembly. The existing bioinformatics tools, for example, a co-evolution detection algorithm called correlated residues of statistical significance (CRoSS) can be used to identify the class of DD pairs straddling two interacting proteins by an evolutionary module^53^, besides, some prediction methods infer protein order in PKS assembly lines, such as PKSpop (co-evolution-based protein-protein interaction prediction algorithm)^70^, DDAP (prediction of the ordering of proteins)^72^, can help to predict and find candidate DDs of specific PKSs for assembly. It should be noted that the DDs pair in the different class could also be assembled in some cases. For example, as the natural assembly DDs, N12 ^C^DD-N13 ^N^DD of nanchangmycin polyketide synthase was classified as *H1a–T1b*^41^. Therefore, phylogenetic clustering analysis based on sequence homology needs to be experimentally explored, with a focus on the assembly of DDs, and the essential mechanism of the DDs needs to be further explored. It’s suggested that the straightforward approach of mPKSeal is to use multiple pairs sourced from the same PKS, which are intrinsically orthogonal interacting, and to combine pairs which evidently belong to distinct structural classes which can be determined. The number of the assembly enzymes is one higher than the number of orthogonal DD pairs. These orthogonal DD pairs can be derived from one natural *cis*-AT PKS or from different phylogenetic classes of DDs originating from different PKSs, or a combination of them (Supplementary Fig. 9).

We developed a strategy named mPKSeal, a synthetic library of enzyme assembly elements formed by DDs of type I *cis*-AT PKSs that assemble key cascade catalysis enzymes while promoting increased target production. Thousands of docking pairs can be used as mPKSeal tools and debottleneck the limited selection of enzyme assembly for different application requirements. This study specifically addresses the challenge of increasing cascade catalytic efficiency, more broadly, highlights the potential for engineering enzyme assembly with available tools. It is envisioned that the strategy can be used as an alternative powerful tool for applications in biocatalysis, metabolic engineering, and synthetic biology.

## Methods

### Materials and reagents

PCR amplification for plasmid construction was performed using Phusion high-fidelity polymerase (M0530S, New England Biolabs, MA, USA) and PrimeSTAR GXL DNA polymerase (R050B, TaKaRa, Kyoto, Japan). PCR primers were synthesized by GENEWIZ (Suzhou, China). All amplified fragments were purified using the D2000 Gel & PCR clean up kit (D2000-00, Omega Bio-Tek, GA, USA). Seamless cloning kits were purchased from Beyotime (D7010S, Beyotime, Shanghai, China). All plasmids were extracted using the TIANpre mini plasmid kit (DP107, TIANGEN, Beijing, China). Fast digest restriction enzymes were purchased from Thermo Fisher Scientific (Indianapolis, IN, USA). Tryptone (FP 318) and yeast extract (FM888) for Luria-Bertani broth (LB) medium were purchased form Angel Yeast Co., Ltd (Wuhan, China). Isopropyl-β-D-thiogalactoside (IPTG) was purchased from Biofroxx GmbH (1122GR001, Einhausen, Germany). Ampicillin sodium (A610028-0025), Kanamycin sulfate (MFCD00070253), and chloramphenicol (MFCD00078159) were purchased form Sangon Biotech Co., Ltd (Shanghai, China). Astaxanthin standard was purchased from Aladdin (A114383, Shanghai, China). All organic reagents for product extraction and HPLC detection were purchased form Sigma-Aldrich (Louis, MO, USA). All inorganic salts were purchased from Sinopharm Chemical Reagent Co., Ltd (Wuhan, China).

### Plasmid and strain construction

Plasmids, strains, and oligonucleotide primers used in this study are listed in Supplementary Data 1, 2, and 3, respectively. Fragments used in plasmid construction were amplified by PCR using the corresponding templates and primers and joined using the Seamless cloning kit (D7010S, Beyotime, Shanghai, China) (Supplementary Data 4). Amino acid sequences of DDs are listed in Supplementary Data 5. *E. coli* DH10B used for cloning and plasmid propagation was cultured on selective LA plates (LB with 1.6% agar) or in selective LB medium. Kanamycin, ampicillin, and chloramphenicol were supplemented at 50, 100, and 34 µg/mL, respectively. All plasmids were verified by digesting with the appropriate restriction enzymes and sequenced. Recombinant *E. coli* BL21(DE3) cells harboring relevant plasmids (Supplementary Data 2) were constructed for astaxanthin production and cell imaging. *E. coli* Rosetta(DE3) cells harboring relevant plasmids were constructed for protein expression.

### Protein expression and purification

The following buffers were used for protein purification: buffer A containing 50 mM Tris-base pH 7.6, 4 mM 2-hydroxy-1-ethanethiol, and 300 mM NaCl; buffer B containing 50 mM Tris-base pH 7.6, 4 mM 2-hydroxy-1-ethanethiol, 300 mM NaCl, and 500 mM imidazole; buffer C containing 50 mM Tris-base pH 7.8 and 50 mM NaCl; and TEV reaction buffer containing 50 mM Tris-base pH 7.8, 50 mM NaCl, 0.5 mM ethylenediaminetetraacetic acid (EDTA), and 1 mM dithiothreitol (DTT). PBS was purchased from Beyotime (C0221A, Beyotime, Shanghai, China).

*E. coli* Rosetta(DE3) containing the protein expression plasmids was cultivated in LB medium with appropriate antibiotics at 37 °C with shaking at 200 rpm until an OD_600_ of 0.6–0.8 was reached. IPTG was added at a final concentration of 0.1 mM, followed by cultivation at 16 °C for 16 h. Cells were centrifuged at 6010 × *g* for 5 min at 4 °C followed by removal of the supernatant. The cell pellet was resuspended in buffer A and lysed by ultrasonication. Lysates were centrifuged at 15,871 × *g* for 30 min at 4 °C and subsequently filtered through a 0.45 μm syringe filter. The supernatant or sediment were detected by SDS-PAGE. Purified proteins were quantified using the Pierce BCA protein assay kit (23225, Thermo Fisher Scientific, IN, USA).

MBP-TEV-D3 ^N^DD-His-tag, MBP-TEV-R5 ^N^DD-His-tag, MBP-TEV-D5 ^N^DD-His-tag, Flag-tag-D2 ^C^DD-TEV-His-tag-MBP-His-tag, Flag-tag-R4 ^C^DD-TEV-His-tag-MBP-His-tag, and Flag-tag-D4 ^C^DD-TEV-His-tag-MBP-His-tag were purified by fast protein liquid chromatography (FPLC) (UEV25D, Union-Biotech Co., Ltd, Shanghai, China) equipped with a HisTrap HP column (5×1 mL) (17524801, GE, MA, USA). The sample buffer was exchanged into TEV reaction buffer following protein concentration and desalting. Proteins were digested using TEV protease (50:1 w/w) at 4 °C with shaking (80 rpm) for 3–5 h to remove the MBP and filtered using a 0.45 μm syringe filter. The filtrates were re-loaded onto a HisTrap HP column and washed with buffer A (ten column volume) and buffer B (ten column volume), and the eluents were collected. The eluents (buffer B) of D3 ^N^DD-His-tag, R5 ^N^DD-His-tag, and D5 ^N^DD-His-tag then purified by FPLC equipped with a Superdex 75 Increase 10/300 GL column (29148721, GE, MA, USA), and the peaks corresponding to the exact size of each DD were collected by comparison with a standard curve of standard protein marker. Meanwhile, the eluents (buffer B) of Flag-tag-D2 ^C^DD, Flag-tag-R4 ^C^DD and Flag-tag-D4 ^C^DD were purified by Anti-Flag Affinity Gel (P2282, Beyotime, Shanghai, China). Samples were detected by 15.5%-Tris-Tricine-SDS-PAGE (P1321, Solarbio, Beijing, China). Following protein concentration and desalting, PBS buffer was exchanged to the work solution of interaction analysis assays. The purified DDs were unstable in solution; therefore, samples were freeze-dried and stored at -80 °C.

### Interaction analysis assays

Bio-layer interferometry equipments (Octet RED96E, ForteBio, Goettingen, Germany and GatorPrime, Gator Bio, CA, USA) were used to determine the C- and N-terminal binding activities. D5 ^N^DD powders were redissolved in PBS buffer and biotinylated according to the manufacturer’s instructions (G-MM-IGT, Genemore, Shanghai, China). Biotinylated samples were diluted to 100 μg/mL with PBS, then immobilized on a SA (Streptavidin) biosensor (19906131, Fortebio, Washington, USA). D3 ^N^DD and R5 ^N^DD powders were redissolved to 100 μg/mL with PBS and immobilized on a Ni-NTA biosensor (20-5009, Gator, Shanghai, China). D2 ^C^DD, D4 ^C^DD and R4 ^C^DD powders were redissolved in 1× kinetic buffer (PBS pH 7.4, 0.1% BSA, and 0.05% Tween 20) at variable concentrations. Interaction assays were performed in 5 steps: 1) baseline acquisition; 2) ^N^DD loading onto sensor; 3) second baseline acquisition; 4) association of ^C^DD for *k*_*a*_ measurement; and 5) dissociation of ^C^DD for *k*_*d*_ measurement. Baseline acquisition and dissociation steps were carried out in 1× kinetic buffer. Octet RED96E was used to test the interaction of D5 ^N^DD and D4 ^C^DD (D2 ^C^DD), the other DDs pairs were performed by GatorPrime. Binding kinetics were determined using the 1:1 global model with Fortebio data analysis software version 11.1 and Gator^TM^ Part11 Software. The binding assays were performed three times.

### Fluorescence microscopy

Confocal images were collected using a Nikon A1R laser scanning confocal microscope (Tokyo, Japan). Single colonies of strains expressing fluorescent proteins were cultured in 5 mL LB medium with appropriate antibiotics at 30 °C with shaking at 200 rpm until an OD_600_ of 0.7–0.9, followed by induction with IPTG at a final concentration of 0.1 mM for 4 h. The cell pellets were collected and microscopy images were obtained using an oil lens. The appropriate laser intensities (Ex: 488 nm for eGFP; Ex: 561 nm for mCherry) were adjusted to remove cross-talk of the fluorescent signals. Images were analyzed with Nikon NIS-Elements Imaging software.

### Shake-flask fermentation of astaxanthin-producing strains

Single colonies of strains were cultured in 5 mL LB medium with appropriate antibiotics and shaken (200 rpm) overnight at 30 °C to obtain the seed culture. For shake-flask fermentation, the seed culture was sub-cultured into 100 mL LB medium with appropriate antibiotics in 500 mL flask by 1% (v:v) at 30 °C, 200 rpm. When the OD_600_ reached 0.7–0.9, cells were induced by 0.1 mM IPTG. Samples (2 mL) were collected after 4 h of induction for further analysis. Three biological replicates were performed starting from different fermentations.

### Analysis assay of astaxanthin

Cells were collected by centrifugation at 15,871 × *g* for 1 min. The cell pellets were extracted with equal volume methanol and acetone (1:4, v/v) by vortexing in the dark until colorless. The extract was centrifuged at 15,871 × *g* for 10 min. The supernatant was filtered using an organic filter (0.22 µm, Nylon, LENG RUI, Nantong, China) and analyzed using an Agilent 1260 HPLC system equipped with a ZORBAX SB 80 Å C18 column (883975-902, 4.6 mm × 150 mm, 5 μm, Agilent Technologies, Inc., CA, USA) at 474 nm and 25 °C. Samples were eluted with the following gradient program consisting of buffer A (H_2_O with 0.1% trifluoroacetic acid) and B (acetonitrile with 0.1% trifluoroacetic acid) at a flow rate of 1.0 mL/min: 0–5 min, 50%–100% B; 5–25 min, 100% B; 25–30 min, 100%–50% B. Astaxanthin was quantified using a standard curve with an appropriate dilution factor and averaged for three replicate analyses. The final production of astaxanthin were calculated relative to the gram weight of the cells.

### Reporting summary

Further information on research design is available in the Nature Research Reporting Summary linked to this article.

## Supplementary information

Supplementary Information

Description of Additional Supplementary Files

Supplementary Data 1

Supplementary Data 2

Supplementary Data 3

Supplementary Data 4

Supplementary Data 5

Reporting Summary

## Data Availability

Data supporting the findings of this work are available within the paper and its Supplementary Information files. A reporting summary for this Article is available as a Supplementary Information file. Source data are provided with this paper.

## Source data

Source Data
