# Supplementary material for: Metabolic pathway assembly using docking domains from type I cis-AT polyketide synthases
Source: Nat Commun. 2022 Sep 21;13:5541. doi: 10.1038/s41467-022-33272-2 (PMC9492657; doi:10.1038/s41467-022-33272-2)
Supplement: Supplementary file 1 — Supplementary Information [file 41467_2022_33272_MOESM1_ESM.docx]

**Metabolic pathway assembly using docking domains from type I *cis*-AT polyketide synthases**

Sun *et al.*

**Supplementary Fig. 1. Determination of wavelength shift (nm) of the interactions of DDs. a-c** Wavelength shift (nm) generated by the addition of D2 ^C^DD, D4 ^C^DD, or R4 ^C^DD at different concentrations to the complementary D3 ^N^DD, D5 ^N^DD, or R5 ^N^DD, respectively, for three replicates. **d,e** Wavelength shift (nm) generated by the addition of D2 ^C^DD or R4 ^C^DD at different concentrations to the non-complementary D5 ^N^DD, or D3 ^N^DD, respectively, for three replicates. Source data are provided as a Source Data file.

**Supplementary Fig. 2**. **Optical microscopy assays of insulativity of undocking DDs from the same PKS systems. a** Confocal microscopy images of cells expressing Idi-mCherry and CrtE-eGFP tagged with undocking D2 ^C^DD-D5 ^N^DD from DEBS. **b** Confocal microscopy images of cells expressing Idi-mCherry and CrtE-eGFP tagged with undocking D4 ^C^DD-D3 ^N^DD from DEBS. **c** Confocal microscopy images of cells expressing Idi-mCherry and CrtE-eGFP tagged with undocking R4 ^C^DD-R11 ^N^DD from RAPS. **d** Confocal microscopy images of cells expressing Idi-mCherry and CrtE-eGFP tagged with undocking R10 ^C^DD-R5 ^N^DD from RAPS. **a-d** Scale bar: 5 μm. Experiments were repeated at least three times with similar results.

**Supplementary Fig. 3**. **The shake-flask fermentation of astaxanthin of unassembled strains. a** The fermentation of control strains containing only one of DD corresponding to di-enzyme assembly of mDEBSeal. The strain A0 was the original control strain without any of DDs. The strains A2 and A3 contained only one of DDs, Idi-D2 ^C^DD and D3 ^N^DD-CrtE, respectively. **b** The fermentation of control strains containing one or two of DDs corresponding to cytoplasmic tri-enzyme assembly of mDEBSeal. The strains A5, A6, and A7 contained AtoB-D2 ^C^DD, D3 ^N^DD-ERG13-D4 ^C^DD, and D5 ^N^DD-tHMG1, respectively. **c** The fermentation of control strains containing one or two of DDs corresponding to cytoplasmic-membranous tri-enzyme assembly of mDEBSeal. The strains A2, A9, and A10 contained Idi-D2 ^C^DD, D3 ^N^DD-CrtE-D4 ^C^DD, and D5 ^N^DD-CrtB, respectively. **d** The fermentation of control strains containing one or two of DDs corresponding to membranous tri-enzymes assembly of mDEBSeal. The strains A12, A13, and A14 contained CrtY-D2 ^C^DD, D3 ^N^DD-CrtZ-D4 ^C^DD, and D5 ^N^DD-CrtW, respectively. **a-d** Experiments were repeated three times, and each point represents the mean ± SD. Statistical analysis was performed using a two-tailed Student’s *t*-test (*****P<*0.0001, ****P<*0.001, **P<*0.1, ns, *P*≥0.05). *P* value in **a**, *P^A2^*=0.5167, *P^A3^*=0.0115. *P* value in **b**, *P^A5^*=0.2689, *P^A6^*=0.2475, *P^A7^*=0.4984. *P* value in **c**, *P^A2^*=0.5167, *P^A9^*<0.0001, *P^A10^*=0.9344. *P* value in **d**, *P^A12^*=0.5098, *P^A13^*=0.0003, *P^A14^*=0.9961. Source data are provided as a Source Data file.

**Supplementary Fig. 4. The products profile of carotenoids in astaxanthin producing strains. a** The accumulation of the main carotenoids in astaxanthin producing strains. **b** The total carotenoids in astaxanthin producing strains. Strain A1 contained the cytoplasmic-membranous di-enzyme assembly of Idi-CrtE tagged with D2 ^C^DD-D3 ^N^DD. Strain A4 contained the cytoplasmic tri-enzyme assembly of AtoB-ERG13-tHMG1 tagged with D2 ^C^DD-D3 ^N^DD and D4 ^C^DD-D5 ^N^DD. Strain A8 contained the cytoplasmic-membranous tri-enzyme assembly of Idi-CrtE-CrtB tagged with D2 ^C^DD-D3 ^N^DD and D4 ^C^DD-D5 ^N^DD. Strain A11 contained a membranous tri-enzyme assembly of CrtY-CrtZ-CrtW tagged with D2 ^C^DD-D3 ^N^DD and D4 ^C^DD-D5 ^N^DD. Strain A0 without DDs acted as the control. **a, b** Empty filled cloud: candidate assembly enzyme; purple filled cloud: AtoB; dark blue filled cloud: ERG13; green filled cloud: tHMG1; blue filled cloud: Idi; orange filled cloud: CrtE; pink filled cloud: CrtB; red filled cloud: CrtY; violet filled cloud: CrtZ; ginger filled cloud: CrtW. Experiments were repeated three times, and each point represents the mean ± SD. Statistical analysis was performed using a two-tailed Student’s *t*-test (*****P<*0.0001, ****P<*0.001, **P<*0.1). *P^A1^*=0.0010, *P^A4^*=0.0476, *P^A8^*<0.0001, *P^A11^*=0.0048. Source data are provided as a Source Data file.

**Supplementary Fig. 5**. **The shake-flask fermentation of astaxanthin of unassembled strains corresponding to mAURSeal, mFKBSeal and mRAPSeal. a** The fermentation of control strains containing one or two of DDs corresponding to Idi-CrtE-CrtB assembly of mAURSeal. The strains A16, A17, and A18 contained Idi-A1 ^C^DD, A2 ^N^DD-CrtE-A2 ^C^DD, and A3 ^N^DD-CrtB, respectively. **b** The fermentation of control strains containing one or two of DDs corresponding to Idi-CrtE-CrtB assembly of mFKBSeal. The strains A20, A21, and A22 contained Idi-F4 ^C^DD, F5 ^N^DD-CrtE-F6 ^C^DD, and F7 ^N^DD-CrtB, respectively. **c** The fermentation of control strains containing one or two of DDs corresponding to Idi-CrtE-CrtB assembly of mRAPSeal. The strains A24, A25, and A26 contained Idi-R4 ^C^DD, R5 ^N^DD-CrtE-R10 ^C^DD, and R11 ^N^DD-CrtB, respectively. **a-c** Experiments were repeated three times, and each point represents the mean ± SD. Statistical analysis was performed using a two-tailed Student’s *t*-test (ns, *P*≥0.05). *P* value in **a**, *P^A16^*=0.8878, *P^A17^*=0.3803, *P^A18^*=0.0782. *P* value in **b**, *P^A20^*=0.9998, *P^A21^*=0.9471, *P^A22^*=0.1807. *P* value in **c**, *P^A24^*=0.4765, *P^A25^*=0.2796, *P^A26^*=0.8811. Source data are provided as a Source Data file.

**Supplementary Fig. 6. The shake-flask fermentation of astaxanthin of unassembled strains corresponding to combinatorial usage of mPKSeal from different *cis*-AT PKSs. a** The strains A28, A29, A24, A31, A33 and A34 expressed single DDs Idi-R10 ^C^DD, D5 ^N^DD-CrtE, Idi-R4 ^C^DD, F5 ^N^DD-CrtE, Idi-S7 ^C^DD and C10 ^N^DD-CrtE, respectively. **b** The strains A16, A37, and A40 expressed single DDs, Idi-A1 ^C^DD, R5 ^N^DD-CrtB and S8 ^N^DD-CrtB, respectively. The strains A36 and A39 expressed two of DDs, A2 ^N^DD-CrtE-R4 ^C^DD and A2 ^N^DD-CrtE-S7 ^C^DD, respectively. The strain A0 represented the control without any of DDs. **a-b** Experiments were repeated three times, and each point represents the mean ± SD. Statistical analysis was performed using a two-tailed Student’s *t*-test (ns, *P*≥0.05). *P* value in **a**, *P^A28^*=0.1196, *P^A29^*=0.5511, *P^A24^*=0.6126, *P^A31^*=0.9962, *P^A33^*=0.7348, *P^A34^*=0.6876. *P* value in **b**, *P^A16^*=0.8734, *P^A36^*=0.8442, *P^A37^*=0.2899, *P^A39^*=0.2099, *P^A40^*=0.8105. Source data are provided as a Source Data file.

**Supplementary Fig. 7. Optical microscopy assays of insulativity of undocking DDs from different PKS DDs clustering. a** Confocal microscopy images of cells expressing Idi-mCherry and CrtE-eGFP tagged with undocking DDs A1 ^C^DD-R5 ^N^DD, in which A1 ^C^DD was from AURS and R5 ^N^DD was from RAPS. **b** Confocal microscopy images of cells expressing Idi-mCherry and CrtE-eGFP tagged with undocking peptides R4 ^C^DD-A2 ^N^DD, in which A2 ^N^DD was from AURS and R4 ^C^DD was from RAPS. **c** Confocal microscopy images of cells expressing Idi-mCherry and CrtE-eGFP tagged with undocking DDs A1 ^C^DD-S8 ^N^DD, in which A1 ^C^DD was from AURS and S8 ^N^DD was from stigmatellin synthase. **d** Confocal microscopy images of cells expressing Idi-mCherry and CrtE-eGFP tagged with undocking DDs S7 ^C^DD-A2 ^N^DD, in which A2 ^N^DD was from AURS and S7 ^C^DD was from stigmatellin synthase. **a-d** Scale bar: 5 μm. Experiments were repeated at least three times with similar results.

**Supplementary Fig. 8. The astaxanthin production comparison between mPKSeal and simple enzyme fusion strategies.** Strain A0 without DDs or fusion linkers acted as the control. Strain A1 contained the cytoplasmic-membranous di-enzyme assembly of Idi-CrtE tagged with D2 ^C^DD-D3 ^N^DD. Strain A41 contained the fusion enzyme CrtE-Idi. Strain A42 contained the fusion enzyme Idi-CrtE. Experiments were repeated three times, and each point represents the mean ± SD. Statistical analysis was performed using a two-tailed Student’s *t*-test (*****P<*0.0001, ns, *P*≥0.05). *P^A1^*<0.0001, *P^A41^*=0.4277, *P^A42^*=0.7125. Source data are provided as a Source Data file.

**Supplementary Fig. 9. Multiple usage of mPKSeal tools.** **a** Multi-enzyme assembly mediated by DDs from natural PKSs. Fkb, tacrolimus PKS; Sti, stigmatellin PKS; Mon, monensin PKS; M, module; E, enzyme; LDD: loading didomain; TE, thioesterase. Red connecting pairs, class I DDs; blue connecting pairs, class II DDs; purple connecting pairs, class III DDs. **b** Combinatorial usage of DDs from different PKS systems based on orthogonal classes.
