# Supplementary material for: Metabolic pathway assembly using docking domains from type I cis-AT polyketide synthases
Source: Nat Commun. 2022 Sep 21;13:5541. doi: 10.1038/s41467-022-33272-2 (PMC9492657; doi:10.1038/s41467-022-33272-2)
Supplement: Supplementary file 2 — Description of Additional Supplementary Files [file 41467_2022_33272_MOESM2_ESM.pdf]

Description of Additional Supplementary Files

File Name: Supplementary Data 1
Description: Plasmids used in this study.

File Name: Supplementary Data 2
Description: Strains used in this study.

File Name: Supplementary Data 3
Description: Primers used in this study.

File Name: Supplementary Data 4
Description: Details of the plasmid construction in this study.

File Name: Supplementary Data 5
Description: Amino acid sequences of DDs used in this study.
